# Phenotypic and metabolic characterization of an *ADH2* deletion mutant associated with reduced fluconazole susceptibility in *Candida albicans*

**DOI:** 10.3389/fcimb.2026.1911561

**Published:** 2026-10-09

**Authors:** Chunjiao Zheng, Wenjing He, Chang Su, Lulu An, Jingwen Tan, Lianjuan Yang

**Affiliations:** 1 Department of Medical Mycology, Center of Infectious Skin Diseases, Shanghai Skin Disease Hospital, Tongji University School of Medicine, Shanghai, China; 2 Hubei Key Laboratory of Cell Homeostasis, College of Life Sciences, Wuhan University, Wuhan, China; 3 Laboratory of Pathogen-Host Interaction (Tongji University), Ministry of Education, Shanghai, China

**Keywords:** ADH2, alcohol dehydrogenase, Candida albicans, fluconazole susceptibility, glucose-metabolism-related transcriptional changes, redox homeostasis

## Abstract

**Introduction:**

Alcohol dehydrogenases (ADHs) participate in fungal carbon metabolism and redox homeostasis, but the contribution of *ADH2* to virulence-associated phenotypes and fluconazole response in *Candida albicans* (*C. albicans*) remains incompletely defined.

**Methods:**

An *ADH2* deletion mutant, *adh2Δ/Δ*, was generated in a SC5314 genetic background. Growth kinetics, adhesion, filamentous development, biofilm metabolic activity, cell surface hydrophobicity, intracellular ATP, reactive oxygen species, mitochondrial membrane potential, NAD^+^/NADH ratio, selected glucose-metabolism-related transcript levels, fluconazole response, and *in vivo* infection burden were evaluated.

**Results:**

The *adh2Δ/Δ* mutant displayed modestly altered growth kinetics and showed a shorter doubling time than SC5314. However, qualitative assays showed reduced adhesion and filamentous development, accompanied by decreased expression of *ALS1*, *ALS3*, and *HWP1*. The mutant displayed increased biofilm metabolic activity but decreased cell surface hydrophobicity, together with increased *CSH1* expression. Metabolic analyses showed reduced ATP levels and basal intracellular ROS-sensitive fluorescence, together with an increased NAD^+^/NADH ratio and elevated mitochondrial membrane potential. Among selected glucose-metabolism-related genes, *HXK1* was significantly upregulated after correction for multiple testing, whereas *CDC19*, *PGI1*, and *PGK1* were not significantly altered. The *adh2Δ/Δ* mutant showed a two-fold increase in fluconazole MIC and stronger growth under FLCZ stress, accompanied by increased *ERG11* transcript levels, whereas *MDR1*, *CDR1*, and *CDR2* were not significantly altered after correction for multiple testing; *ADH1* was also unchanged. In mice, *adh2Δ/Δ* infection resulted in higher renal fungal burden and higher residual renal fungal burden after fluconazole treatment.

**Discussion:**

These findings define an *ADH2* deletion-associated phenotypic and metabolic/redox state and provide a hypothesis-generating framework for exploring its association with reduced fluconazole susceptibility in *C. albicans*.

## 1 Introduction

Invasive fungal infections (IFIs) have become a major global health challenge, particularly among immunocompromised populations, including patients undergoing chemotherapy, organ transplant recipients, and individuals living with HIV/AIDS (Denning, 2024; Lass-Flörl et al., 2024). Recent global estimates suggest that invasive fungal infections affect approximately 6.5 million people annually and are associated with approximately 3.8 million deaths worldwide (Denning, 2024). Among these infections, invasive candidiasis represents a major clinical burden (Lass-Flörl et al., 2024; Parambath et al., 2024). *Candida albicans (C. albicans)* remains one of the leading causes of invasive candidiasis worldwide and is associated with substantial mortality, although the distribution of *Candida* species varies according to geographic region, patient population, and antifungal exposure history (Lass-Flörl et al., 2024; Parambath et al., 2024). Fluconazole (FLCZ) remains an important antifungal agent for the treatment and prevention of *Candida* infections; however, acquired azole resistance and reduced susceptibility have been increasingly recognized in clinical settings, complicating antifungal management (Rogers et al., 2022; Parambath et al., 2024). These challenges highlight the need to identify new mechanisms linking *C. albicans* pathogenicity, metabolic adaptation, and reduced antifungal susceptibility.

Alcohol dehydrogenases (ADHs) are a broad family of oxidoreductases widely distributed across bacteria, fungi, plants, and mammals. They catalyze the reversible oxidation of alcohols to aldehydes or ketones, including the interconversion between ethanol and acetaldehyde, thereby contributing to carbon metabolism and cellular redox homeostasis (de Smidt et al., 2012; Gutiérrez-Corona et al., 2023). In fungi, ADH enzymes participate in aerobic and anaerobic growth, morphogenetic processes, stress adaptation, and pathogenicity, and the number and function of ADH family members vary among fungal species (Gutiérrez-Corona et al., 2023). In *Saccharomyces cerevisiae* (*S. cerevisiae*), classical ethanol metabolism involves multiple ADH isoenzymes. Adh1 primarily catalyzes the reduction of acetaldehyde to ethanol during glucose fermentation, accompanied by oxidation of NADH to NAD^+^, whereas the glucose-repressed isoenzyme Adh2 mainly participates in ethanol utilization and catalyzes ethanol oxidation to acetaldehyde with reduction of NAD^+^ to NADH under appropriate metabolic conditions (de Smidt et al., 2012). In *C. albicans*, several ADH homologs, including *ADH1*, *ADH2*, and *ADH3*, have been examined in the context of acetaldehyde metabolism, but the functional contribution of individual ADH members remains incompletely defined (Bakri et al., 2015). Recent comparative analyses further indicate that ADH family members are involved in fungal development, biofilm formation, pathogenicity, and fluconazole response in pathogenic fungi, supporting the potential importance of ADH-associated metabolic regulation in fungal adaptation (Liu et al., 2025). These observations provide a rationale for investigating whether *C. albicans ADH2* contributes to virulence-associated phenotypes, metabolic reprogramming, and fluconazole response.

Among the ADH family members in *C. albicans*, *ADH1* has been relatively more extensively studied than *ADH2. ADH1* encodes alcohol dehydrogenase 1 and has been implicated in ethanol and acetaldehyde metabolism; *in vitro* analyses showed constitutive *ADH1* expression under multiple growth conditions and suggested that Adh1 contributes to ethanol-utilizing, acetaldehyde-producing activity in *C. albicans* (Bakri et al., 2015). Recent functional work further linked Adh1 to biological traits and pathogenicity-associated processes in *C. albicans* (Wang et al., 2025). In addition, *ADH1* has been associated with azole-resistance-related gene expression. In isolates from patients with vulvovaginal candidiasis, *ADH1* expression correlated with *CDR1*, *CDR2*, and *FLU1* expression (Guo et al., 2013), whereas Siikala et al. reported an inverse correlation between *ADH1* and *CDR1/CDR2* expression in oral isolates from patients with autoimmune polyendocrinopathy-candidiasis-ectodermal dystrophy (Siikala et al., 2011). These apparently divergent findings suggest that the relationship between ADH-linked metabolism and azole-resistance-associated gene expression may depend on host niche, isolate background, and environmental conditions.

Compared with *ADH1*, research on *ADH2* in *C. albicans* remains limited. Previous studies of *C. albicans* alcohol dehydrogenase genes have mainly examined their expression in acetaldehyde metabolism and oral colonization-associated settings. *In vitro* analyses showed that *CaADH1* is constitutively expressed under multiple growth conditions, whereas *CaADH2* displays differential expression; however, CaAdh2p did not show detectable ethanol-utilizing activity in recombinant expression assays, and CaAdh1p was proposed to be the major enzyme responsible for ethanol use under the tested conditions (Bakri et al., 2015). Detection of *ADH1* and *ADH2* transcripts in human oral biopsy samples further suggested that the biological role of *ADH2* remains incompletely defined (Bakri et al., 2014). Although fungal ADH enzymes are increasingly recognized as contributors to metabolism, redox homeostasis, stress adaptation, and pathogenicity (Gutiérrez-Corona et al., 2023), whether *C. albicans ADH2* loss is associated with changes in morphogenetic switching, adhesion, biofilm formation, and fluconazole response has not been systematically investigated. Therefore, whether *ADH2*-associated metabolic changes are linked to pathogenicity-related phenotypes and fluconazole response in *C. albicans* remains an unresolved question.

By focusing on *ADH2*, this study addresses an underexplored metabolic node that may connect ethanol/acetaldehyde-associated redox metabolism with virulence-associated adaptation and fluconazole response in *C. albicans*. Because ADH enzymes participate in NAD^+^/NADH-dependent carbon metabolism, *ADH2* deletion may be associated with changes in intracellular redox balance and glucose-metabolism-related transcriptional responses, thereby providing a rationale for examining metabolic and fluconazole-response phenotypes. In this context, *ADH2* provides a potentially important entry point for understanding how metabolic adaptation contributes to reduced fluconazole susceptibility beyond canonical resistance mechanisms. Therefore, this study aimed to characterize the *ADH2* deletion-associated phenotype across three related domains: virulence-associated cellular phenotypes, metabolic/redox and glucose-metabolism-related transcriptional changes, and fluconazole/*in vivo* infection-response phenotypes. These observations were integrated into a hypothesis-generating framework without assuming a linear causal relationship among these domains.

## 2 Materials and methods

### 2.1 Construction and growth conditions of the *adh2Δ/Δ* mutant

The *C. albicans* parental control strain used in this study was maintained in the SC5314 genetic background. For simplicity, this parental control strain is referred to as SC5314 throughout the manuscript and figures; detailed strain identity and genotype information are provided in **Supplementary Table S1** in the Supporting Information. The homozygous *ADH2* deletion mutant, *adh2Δ/Δ*, was constructed through fusion PCR and homologous recombination as described previously (Hernday et al., 2010). Briefly, the upstream and downstream homologous arms of *ADH2* and the selection marker fragments were amplified separately. The deletion cassette was generated by fusion PCR and transformed into the parental control strain to replace the *ADH2* coding region through homologous recombination. Positive transformants were selected on appropriate selective media and verified by PCR amplification. The primers and strain construction information are provided in **Supplementary Tables S1**–**S4** and **Supplementary Figures S1**–**S5** in the **Supplementary Materials**. Unless otherwise stated, all strains were cultured in yeast extract–peptone–dextrose (YPD) broth or on YPD agar.

### 2.2 Growth kinetics

Strains were separately cultured in yeast extract-peptone-dextrose (YPD) liquid medium. Cells in the late exponential growth phase were harvested by centrifugation, washed twice with sterile phosphate-buffered saline (PBS), and then resuspended in YPD liquid medium. The concentration of the yeast suspension was adjusted to an optical density at 600 nm (OD_600_) = 0.04. The obtained suspension was placed in a 250-mL Erlenmeyer flask and cultured at 30 °C with shaking at 200 rpm. The OD_600_ value of each yeast suspension was measured at 0, 2, 4, 6, 8, 10, 12, 24, and 30 h to plot the growth curve of the strain. Three independent culture flasks were used for each strain within each experiment. The doubling time (DT) during the logarithmic growth phase was calculated separately for each independent culture based on yeast cell concentrations determined by manual counting with a hemocytometer, using the formula: DT = t × lg2/lg [Nt/N0], where N0 and Nt represent the yeast cell concentrations at the beginning and end of the selected logarithmic-growth interval, respectively, and t represents the duration of this interval. The 8–12 h interval was selected for calculation because both strains were within the logarithmic growth phase during this period (Song et al., 2019).

A spot-growth assay was performed to qualitatively compare the growth capacity of SC5314 and *adh2Δ/Δ* under the tested condition (Song et al., 2019). *C. albicans* strains were cultured in YPD liquid medium at 30 °C with shaking at 200 rpm overnight. The cells were collected by centrifugation, washed twice with sterile PBS, and resuspended; the concentration of yeast cells was adjusted to 1.0 × 10^6^ cells/mL. The yeast suspension was 10-fold serially diluted in sterile PBS to prepare five stock suspensions. A total of 5 μL of each stock suspension was plated on the surface of YPD. The plates were cultured at 30°C for 48 h before observing the growth of colonies.

### 2.3 Adhesion assay

Adhesion was assessed using a previously described visual adhesion assay with minor modifications (Song et al., 2019). The 12-well flat-bottomed pre-sterilized microtiter plates were incubated with fetal bovine serum overnight at 37°C. A suspension of 1 × 10^7^ *C. albicans* cells in Spider medium was added to the wells and incubated at 37°C for 2 h. Non-adherent cells were removed by washing with PBS. Fresh Spider medium was added to the corresponding wells, and the plates were incubated at 37°C for 24 h. The wells were photographed. The experiment was performed at least three times independently. This assay was used for representative visual comparison of adhesion phenotypes rather than quantitative measurement of adherent biomass or adherent CFU.

### 2.4 Filamentation assay

Filamentous development was assessed under four commonly used and compositionally distinct *C. albicans* morphogenesis-inducing or morphogenesis-supportive conditions, including YPD + 10% fetal bovine serum (YPD + 10% FBS), Spider medium, Lee’s medium, and synthetic low-ammonium dextrose (SLAD) medium, based on previously described *C. albicans* filamentation assay conditions (Song et al., 2019). These media were selected to evaluate whether the morphological phenotype was reproducible across different filamentation-supportive environmental conditions. For liquid induction, cell suspensions of each strain were adjusted to 1 × 10^6^ cells/mL in YPD + 10% FBS, Spider, Lee’s, or SLAD medium, added to 12-well flat-bottom sterile microtiter plates, and incubated at 37 °C for 3 h. Cells were then examined and photographed under a light microscope. Each experiment was performed at least in triplicate. This assay was used for representative visual comparison of filamentation phenotypes rather than quantitative measurement of hyphal length or filamentation frequency.

### 2.5 Biofilm metabolic activity assay

Biofilm-associated metabolic activity was assessed using XTT reduction assay, according to previously described protocols (Song et al., 2019). This assay was used to evaluate XTT-based metabolic activity of biofilm-associated cells rather than total biofilm biomass or biofilm architecture. *C. albicans* was cultured in YPD liquid medium overnight at 37°C with shaking at 150 rpm. The cells were harvested by centrifugation, washed twice with a sterile PBS solution, and resuspended in RPMI 1640 medium to prepare a suspension with a yeast cell concentration of 1 × 10^6^ cells/mL. 100 μL of a suspension of *C. albicans* was added to the wells of 96-well flat-bottomed pre-sterilized microtiter plates, which were prepared with fetal bovine serum in advance and incubated at 37°C for 90 min. Non-adherent cells were removed by washing with PBS. Fresh RPMI 1640 medium was added to the corresponding wells, and the plates were incubated at 37°C for 48 h. The suspension was removed by washing with PBS. 150 μL of XTT was added to the corresponding wells, and the plates were incubated at 37°C for 4 h. 70 μL of suspension was transferred to a new 96-well flat-bottomed pre-sterilized microtiter plate and measured by a microplate reader. Each experiment was performed at least in triplicate.

### 2.6 Cell surface hydrophobicity assay

A microbial adhesion test to hydrocarbon was used to measure the adhesion ability of each strain to a strongly hydrophobic cyclohexane and xylene medium (Song et al., 2019). *C. albicans* was cultured in YPD liquid medium overnight at 37°C with shaking at 150 rpm. The cells were harvested by centrifugation, washed twice with a sterile PBS solution, and resuspended in PBS to prepare 3 mL of a suspension with a yeast cell concentration of OD_600_ = 1.0. After adding 150 μL of cyclohexane and xylene, the suspension was transferred to a glass test tube and rinsed with an acidic solution. The mixture was incubated at 30°C for 10 min, vortexed for 60 s, and then allowed to stand at room temperature for 20 min for phase separation. For OD_600_ measurement, the aqueous phase at the bottom of the tube was quickly withdrawn with a sterile injection needle. The yeast suspension without the addition of cyclohexane and xylene was used as the negative control. CSH (%) was calculated as follows: (OD_600_control - OD_600_experiment)/OD_600_control × 100%. This percentage reflects the relative decrease in cell density in the aqueous phase after hydrocarbon partitioning and was used as an operational measure of cell surface hydrophobicity; it does not represent a direct count of cells within the hydrocarbon phase. Each experiment was repeated three times.

### 2.7 ATP, ROS, and mitochondrial membrane potential assays

All strains were grown in YPD overnight at 30°C. For determination of intracellular ATP concentrations, an aliquot of 1.0 × 10^6^ cells from each strain was mixed with the same volume of BacTiter-Glo™ reagent (Promega Corporation, Madison, WI, USA) and incubated for 5 min at room temperature as described previously (Song et al., 2019). Basal intracellular ROS-sensitive fluorescence was measured in cells grown without fluconazole exposure using the oxidation-sensitive fluorescent dye DCFDA (Song et al., 2019). This assay reflects general intracellular DCFDA-sensitive oxidative activity and was not designed to specifically measure mitochondrial ROS. Briefly, the fungal suspension was adjusted to 2.5 × 10^6^ cells/mL and incubated at 35 °C with shaking at 200 rpm for 6 h. Mitochondrial membrane potential (ΔΨm) was measured using a JC-1 assay kit (GMS10013.5, USA) following the manufacturer’s instructions. Fluorescence was measured using a multimode microplate reader (Thermo Scientific) with excitation at 490 nm. Red fluorescence (590 nm) and green fluorescence (530 nm) were recorded simultaneously. ΔΨm was expressed as the red/green fluorescence ratio (Song et al., 2019).

### 2.8 NAD^+^/NADH ratio assay

For the NAD^+^/NADH ratio calculation, all strains were grown in YPD at 30°C. Logarithmically growing cells were washed three times with prewarmed sterile PBS. Equivalent numbers of fungal cells were collected from each experimental group before lysis to ensure cell-number-matched samples for NAD^+^/NADH measurement. The NADH and NAD^+^ contents were measured using the NAD^+^/NADH Assay Kit (WST-8 Colorimetric Method) (Beyotime, China). Absorbance was measured on a full-wavelength multifunctional enzyme marker instrument (Thermo Scientific). The NAD^+^/NADH ratio was calculated from these cell-number-matched samples. All experiments were performed in triplicate on two separate days.

### 2.9 Quantitative real-time polymerase chain reaction assay

Overnight cultures of tested strains in 5 mL of YPD at 30°C were collected, then diluted into 100 mL of YPD or Spider medium to obtain an OD_600_ of 0.2 and incubated at 37°C for an additional 6 h. Total RNA was extracted using a yeast RNA extraction kit (Jianshi Bio, China) following the manufacturer’s instructions. cDNA was synthesized, and RT-qPCR was done as previously described (Song et al., 2019). *18S rRNA* was used as the internal reference gene for normalization. The raw Ct values of *18S rRNA* were reviewed and showed no systematic differences between the compared groups under the corresponding experimental conditions. The results were analyzed using the 2^−ΔΔCt method. The primer sequences of genes used in the RT-qPCR are shown in **Supplementary Table S5**.

### 2.10 Fluconazole susceptibility testing and spot-growth assay

Fluconazole susceptibility was determined using the broth microdilution method according to the CLSI M27 reference method for antifungal susceptibility testing of yeasts. Briefly, SC5314 and *adh2Δ/Δ* were cultured overnight in YPD liquid medium at 30 °C with shaking at 200 rpm. Cells were harvested, washed twice with sterile PBS, and resuspended in RPMI 1640 medium. The final inoculum concentration was adjusted to 1–3 × 10³ CFU/mL. Serial two-fold dilutions of fluconazole were prepared in RPMI 1640 medium in 96-well microdilution plates. After inoculation, the plates were incubated at 35 °C for 24 h. The fluconazole MIC was defined as the lowest drug concentration that produced a prominent reduction in growth, corresponding to approximately 50% growth inhibition, compared with the drug-free growth control. Experiments were performed independently three times, and the modal MIC value was reported.

A spot growth assay was used to compare growth under fluconazole stress. *C. albicans* strains were cultured overnight in YPD liquid medium at 30 °C with shaking at 200 rpm. Cells were harvested by centrifugation, washed twice with sterile PBS, and resuspended. The yeast suspension was adjusted to 1.0 × 10^6^ cells/mL. Serial 10-fold dilutions were prepared in sterile PBS to generate five dilutions. A total of 4 μL of each dilution was spotted onto YPD agar plates and YPD agar plates containing 2 μg/mL FLCZ. Plates were incubated at 30 °C for 48 h, and colony growth was observed. Experiments were repeated three times.

### 2.11 Animals

Forty-five specific pathogen-free female ICR mice aged 6–8 weeks and weighing 18–22 g were purchased from Shanghai Jiesijie Laboratory Animal Co., Ltd. All animal experiments were performed in the animal facility of Shanghai Skin Disease Hospital. All animal procedures were approved by the Ethics Committee of Shanghai Skin Disease Hospital, China (approval number: 2020-004). Mice were monitored daily for body weight, survival, general appearance, mobility, and moribund status. Humane endpoints were defined by predefined clinical criteria, including marked progressive body-weight loss, inability or marked difficulty accessing food or water, severe impairment of mobility, severe distress, or a moribund condition. Mice meeting any humane endpoint were euthanized immediately and recorded as events in the survival analysis. Animals reaching humane endpoints or the scheduled experimental endpoint were humanely euthanized by intraperitoneal administration of an overdose of pentobarbital sodium (200 mg/kg body weight). Complete loss of consciousness was confirmed prior to death. All efforts were made to minimize animal suffering.

### 2.12 Evaluation of *in vivo* infection burden and fluconazole treatment response in mice

A total of 45 mice were enrolled in the animal experiment. Eight mice served as noninfected normal controls, whereas the remaining 37 mice underwent fungal inoculation. Because successful establishment of infection could not be guaranteed in every inoculated animal, additional mice were prospectively inoculated to ensure that a sufficient number of successfully infected animals would be available for subsequent treatment and endpoint analyses. Two mice did not meet the predefined criteria for successful infection and were excluded before treatment initiation. Mice were assigned to the SC5314 or *adh2Δ/Δ* infection cohorts before fungal inoculation. Among successfully infected animals within each strain cohort, eligible mice were randomly selected and allocated to the prespecified vehicle- or FLCZ-treatment groups before treatment initiation. Three additional successfully infected animals were retained as reserve animals and were not included in the prespecified analytical cohorts. A total of 40 mice were included in the final analysis, comprising five groups: normal control, SC5314 infection, SC5314 infection plus FLCZ, *adh2Δ/Δ* infection, and *adh2Δ/Δ* infection plus FLCZ, with eight mice in each group. No formal *a priori* sample-size calculation was performed for this exploratory animal experiment. In each group, three mice were assigned to renal fungal-burden and histopathological analyses, and five mice were assigned to survival analysis. No animals were excluded after treatment initiation. Reserve animals not entering the prespecified analytical cohorts were subsequently managed according to the approved animal protocol.

Mice were immunosuppressed with cyclophosphamide at a dose of 100 mg/kg once daily by intraperitoneal injection for 4 days. On the third day of cyclophosphamide treatment, mice were intraperitoneally inoculated with 200 μL of freshly prepared *C. albicans* suspension at 5.0 × 10^7^ cells/mL. At 24 h after fungal inoculation, infected mice were treated with fluconazole at 2 mg/kg once daily by intraperitoneal injection for 4 days. This dose was selected to enable comparative assessment of the *in vivo* fluconazole response between SC5314 and *adh2Δ/Δ* under the same treatment conditions. Fluconazole was first dissolved in DMSO to prepare a 100 mg/mL stock solution and diluted in sterile saline before administration. Another group of infected mice was treated with a vehicle solution as the control. Mouse body weight and survival were monitored. Surviving mice received cyclophosphamide at 75 mg/kg by intraperitoneal injection every other day. Mice showing moribund appearance or reaching humane endpoints were humanely euthanized as described above, and survival curves were recorded. Investigators performing renal CFU counting and histopathological assessment were not blinded to group allocation.

### 2.13 Histopathological analysis of mouse kidneys

For renal fungal-burden and histopathological analyses, three mice per group were euthanized at 24 h after the last FLCZ or vehicle administration. One kidney was isolated, rinsed with sterile saline, weighed, and aseptically homogenized in 1 mL of 0.9% sterile saline. Serial dilutions of the kidney homogenates were plated on SDA medium and incubated for 48 h. Renal fungal burden was calculated on the basis of colony counts and individual kidney weight and expressed as log10 CFU/g kidney. The contralateral kidney was fixed in 4% paraformaldehyde for 24 h and embedded in paraffin. Tissue sections were cut at a thickness of 4 μm. Sections were dewaxed, rehydrated, and stained with hematoxylin and eosin (H&E) or periodic acid–Schiff (PAS). Sections were scanned and photographed at the same magnification. Histopathological images were used for representative qualitative assessment, and no blinded quantitative histopathological scoring was performed.

### 2.14 Statistical analysis

Statistical analyses were performed using GraphPad Prism 10.1. Data are presented as mean ± SD unless otherwise indicated. For single comparisons between two independent groups, an unpaired two-tailed Student’s t-test was used. For RT-qPCR panels in which multiple genes were evaluated within the same biological question, individual between-group comparisons were performed using unpaired two-tailed Student’s t-tests, and P values were adjusted for multiple testing within each panel using the Holm–Šídák method. Growth curves were analyzed using two-way repeated-measures ANOVA, with time treated as the repeated factor and Geisser–Greenhouse correction applied. Renal fungal-burden data from the four infected groups were analyzed using ordinary one-way ANOVA followed by Tukey’s multiple-comparisons test. The normal-control group, in which no fungal colonies were detected, was not included in the log10-transformed fungal-burden analysis. Survival curves were compared using the log-rank (Mantel–Cox) test. Exact P values or multiple-comparison-adjusted P values are reported where available. P < 0.05 was considered statistically significant.

## 3 Results

### 3.1 *adh2Δ/Δ* displays modestly altered basal growth kinetics without growth impairment in *C. albicans*

To characterize basal growth in the *adh2Δ/Δ* mutant, we compared the growth kinetics of SC5314 and the *adh2Δ/Δ* mutant under YPD culture conditions. OD_600_-based growth-curve analysis showed a modest but significant difference in growth kinetics between SC5314 and *adh2Δ/Δ*, with higher OD_600_ values observed for *adh2Δ/Δ* during the later growth phase (strain × time interaction, P < 0.0001; **Figure 1A**). The spot-growth assay showed that *adh2Δ/Δ* retained robust growth capacity on YPD agar (**Figure 1B**). Doubling-time analysis further showed that *adh2Δ/Δ* had a shorter doubling time than SC5314 during the logarithmic growth phase (**Figure 1C**). These results indicate that *adh2Δ/Δ* displays modestly altered basal growth kinetics but does not exhibit a basal growth defect under the tested conditions.

**Figure 1:**
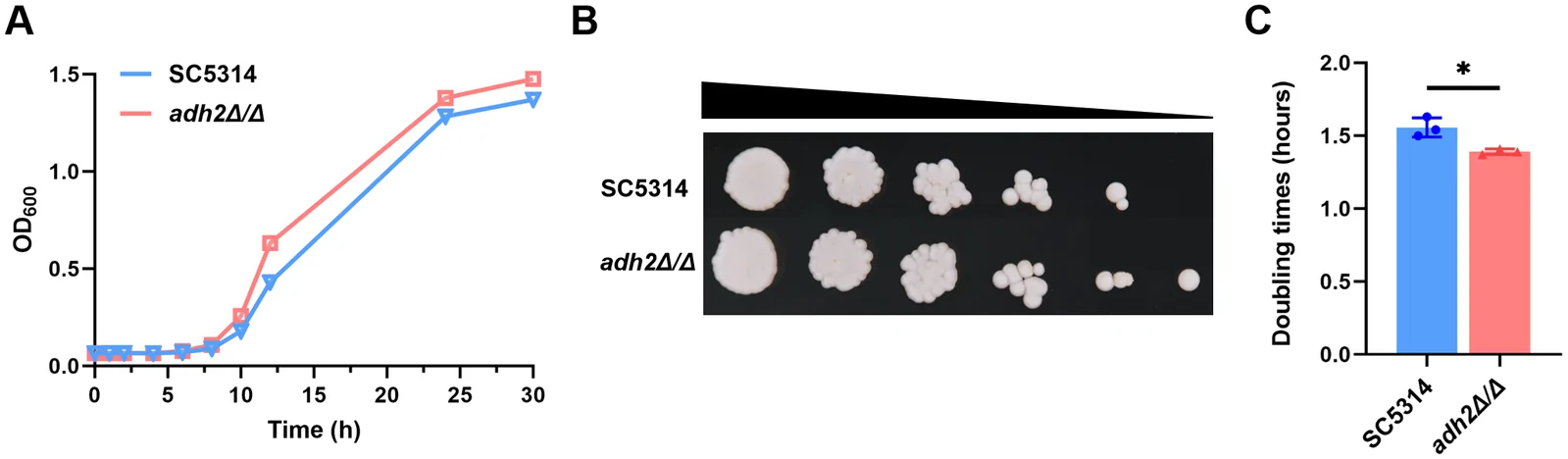
The *adh2Δ/Δ* mutant displays modestly altered basal growth kinetics in *Candida albicans*. **(A)** OD_600_-based growth curves of SC5314 and *adh2Δ/Δ* (n = 3 independent cultures per strain; two-way repeated-measures ANOVA); **(B)** Spot-growth assay on YPD agar; **(C)** Doubling time during the 8–12 h logarithmic-growth interval (n = 3 independent cultures per strain; unpaired two-tailed Student’s t-test, P = 0.0142). *P<0.05.

### 3.2 *adh2Δ/Δ* shows impaired adhesion and filamentous development

We next examined adhesion-related phenotypes in the *adh2Δ/Δ* mutant. In the microtiter plate adhesion assay, representative images showed apparently reduced surface-associated fungal cells in *adh2Δ/Δ* compared with SC5314 (**Figure 2A**). Consistent with this visual observation, the adhesion-associated genes *ALS1* and *ALS3* were significantly downregulated in *adh2Δ/Δ* (**Figure 2B**). We further evaluated filamentous development under liquid induction conditions, including Spider, SLAD, Lee’s, and YPD + 10% FBS. Under these conditions, representative microscopic images showed an apparent reduction in filamentous development in *adh2Δ/Δ* compared with SC5314 (**Figure 2C**). This morphological observation was accompanied by significant downregulation of the hypha-associated gene *HWP1* in *adh2Δ/Δ* (**Figure 2D**). Together, these qualitative phenotypic observations and transcriptional changes suggest that the *adh2Δ/Δ* mutant is associated with reduced adhesion and filamentous development in *C. albicans*.

**Figure 2:**
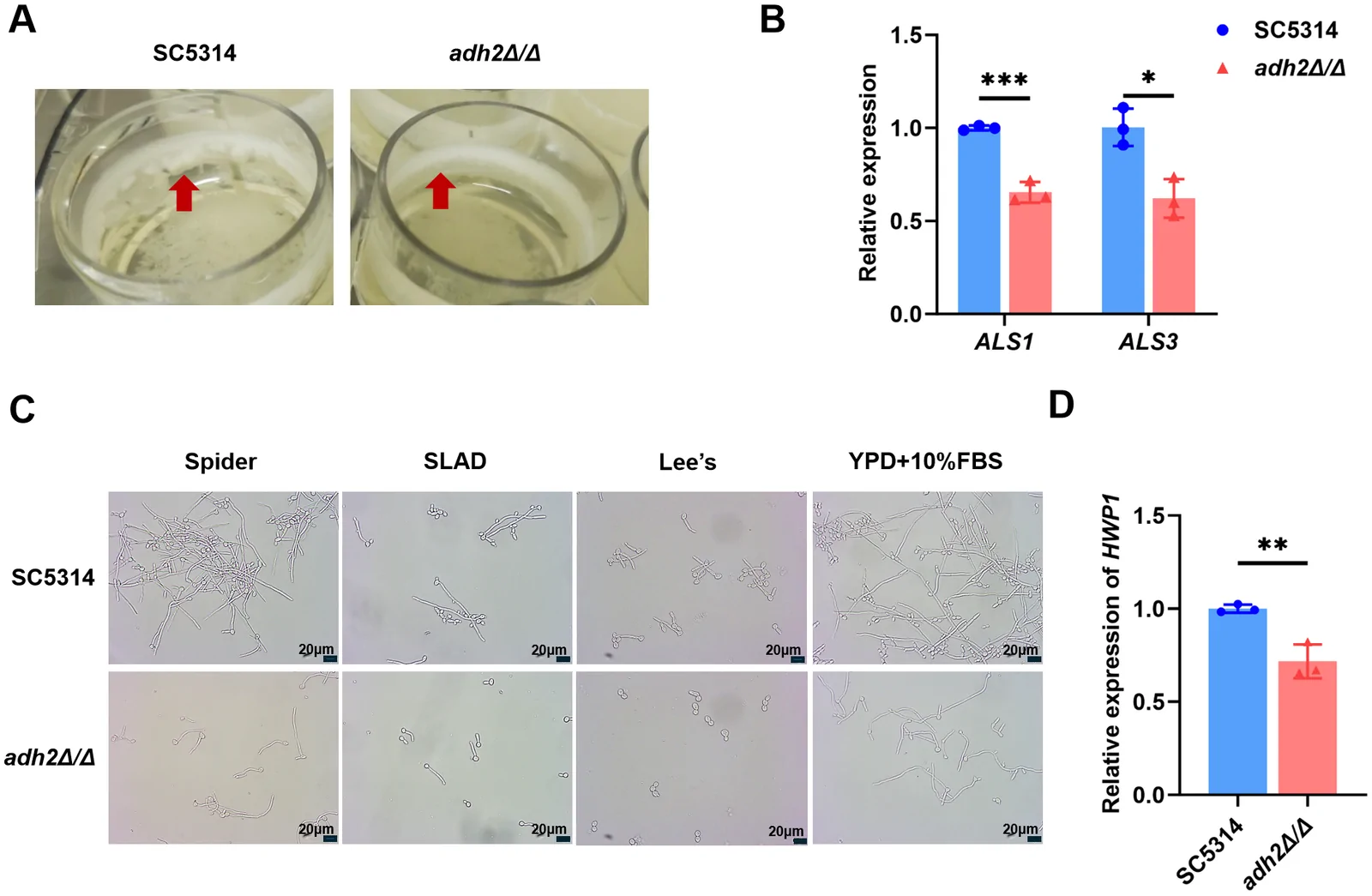
Adhesion- and filamentation-associated phenotypes of the *adh2Δ/Δ* mutant in *Candida albicans*. **(A)** Representative images from the qualitative adhesion assay. Red arrows indicate representative surface-associated fungal material remaining on the sidewall of the plate. **(B)** Relative expression levels of the adhesion-associated genes *ALS1* and *ALS3*. Between-group comparisons were performed using unpaired two-tailed Student’s t-tests, with P values adjusted across the two genes using the Holm–Šídák method (*ALS1*, adjusted P = 0.00094; *ALS3*, adjusted P = 0.0102). **(C)** Representative microscopic images of filamentous development in liquid Spider, SLAD, Lee’s, and YPD + 10% FBS media. Scale bars, 20 μm. **(D)** Relative expression level of the hypha-associated gene *HWP1*. *P<0.05, **P<0.01, ***P<0.001.

### 3.3 *adh2Δ/Δ* exhibits increased biofilm metabolic activity but reduced cell surface hydrophobicity

We further assessed biofilm metabolic activity and cell surface properties. The XTT reduction assay showed that *adh2Δ/Δ* had significantly higher XTT-based biofilm metabolic activity than SC5314 (**Figure 3A**). Because XTT reduction reflects metabolic activity rather than total biofilm biomass, this result should not be interpreted as direct evidence of increased biofilm formation. In contrast, the microbial adhesion to hydrocarbons assay showed that the cell surface hydrophobicity of *adh2Δ/Δ* was markedly decreased (**Figure 3B**). Notably, despite the reduction in cell surface hydrophobicity, the expression of *CSH1* was modestly but significantly increased in *adh2Δ/Δ* (**Figure 3C**). These results indicate that the *adh2Δ/Δ* mutant displays increased XTT-based biofilm metabolic activity but reduced cell surface hydrophobicity, suggesting a dissociation between biofilm-associated metabolic activity and surface-associated properties under the tested conditions.

**Figure 3:**
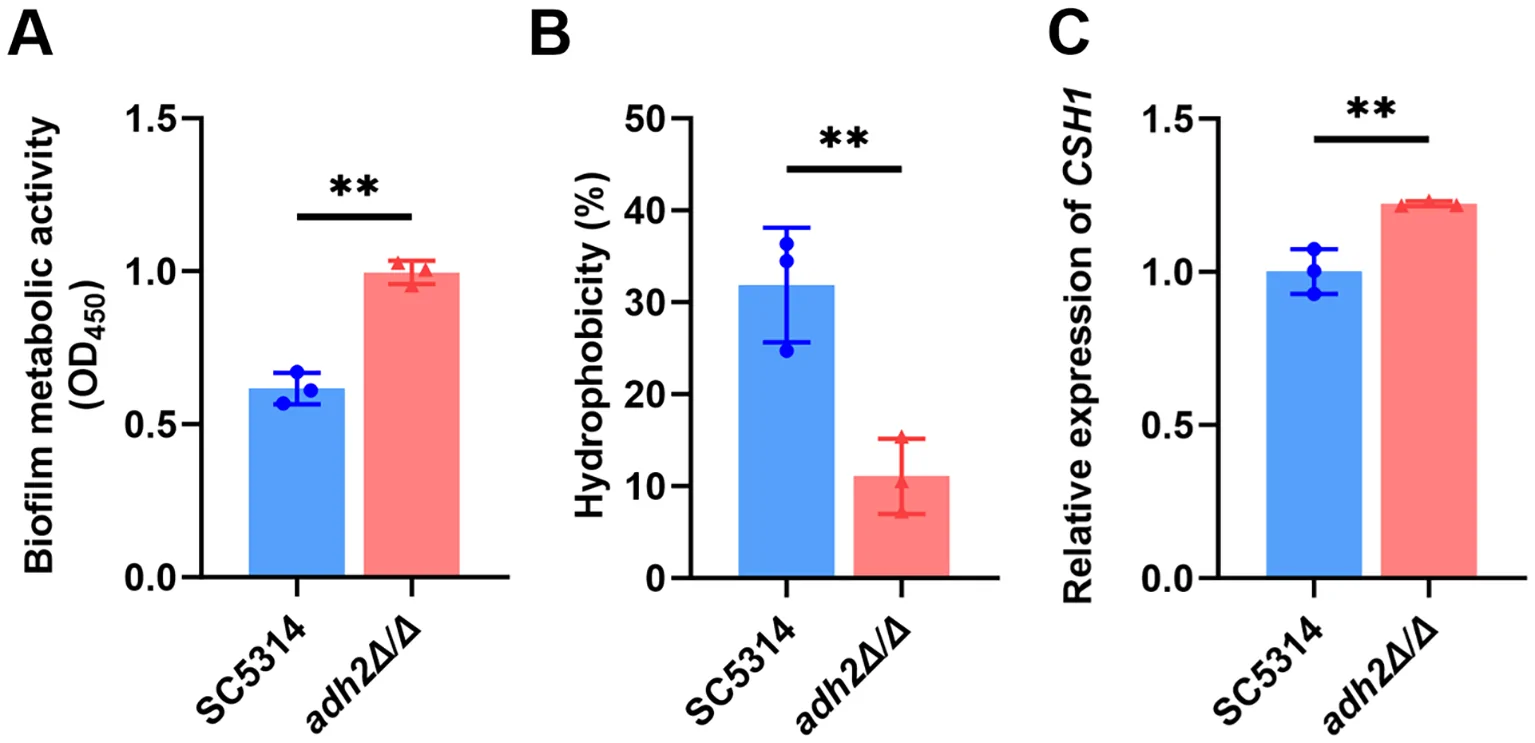
The *adh2Δ/Δ* mutant displays increased XTT-based biofilm metabolic activity and altered cell surface properties. **(A)** XTT-based biofilm metabolic activity; **(B)** cell surface hydrophobicity; **(C)** relative expression level of the cell surface hydrophobicity-associated gene *CSH1*. **P<0.01.

### 3.4 *adh2Δ/Δ* is associated with altered ATP levels, redox status, and mitochondrial membrane potential

Given that Adh2 is an annotated alcohol dehydrogenase family member potentially linked to carbon metabolism and redox homeostasis, we examined the metabolic state of the *adh2Δ/Δ* mutant. Compared with SC5314, *adh2Δ/Δ* showed significantly reduced intracellular ATP levels, indicating a lower intracellular ATP pool (**Figure 4A**). At the same time, *adh2Δ/Δ* exhibited increased mitochondrial membrane potential, decreased basal intracellular ROS-sensitive fluorescence, and an increased NAD^+^/NADH ratio (**Figures 4B–D**). These concurrent changes indicate that the *adh2Δ/Δ* mutant is characterized by altered ATP levels, redox status, and mitochondrial membrane potential. Further analysis of selected glucose-metabolism-related genes showed that *HXK1* was significantly upregulated (P = 0.0181) in *adh2Δ/Δ* after correction for multiple testing, whereas *CDC19*, *PGI1*, and *PGK1* were not significantly altered (**Figure 4E**). Notably, *PGK1* expression showed relatively large inter-replicate variability, and therefore no consistent directional change in *PGK1* expression could be inferred from these data. These results suggest that the *adh2Δ/Δ* phenotype is associated with selective changes in glucose-metabolism-related transcript levels, rather than direct evidence of altered glycolytic flux or uniform upregulation of the glycolytic pathway.

**Figure 4:**
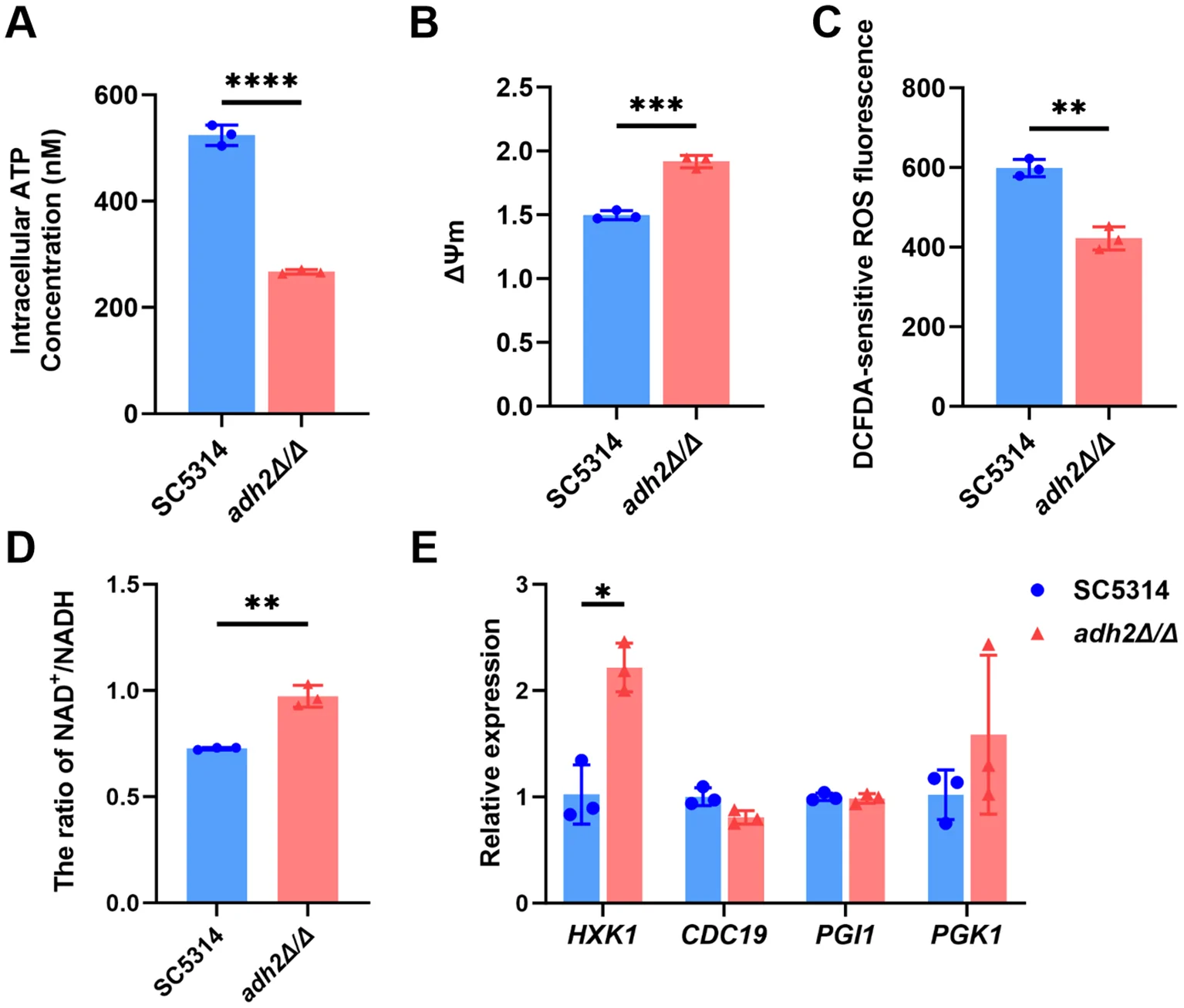
The *adh2Δ/Δ* mutant is associated with altered ATP levels, redox status, and mitochondrial membrane potential in *Candida albicans.* **(A)** Intracellular ATP levels, P < 0.0001; **(B)** mitochondrial membrane potential, represented by the red/green fluorescence ratio, P = 0.0003; **(C)** basal intracellular DCFDA-sensitive ROS fluorescence, P = 0.0011; **(D)** NAD^+^/NADH ratio, P = 0.0012; **(E)** relative expression levels of selected glucose-metabolism-related genes. Expression of each gene was normalized independently to the corresponding SC5314 control. Between-group comparisons were performed using unpaired two-tailed Student’s t-tests, with P values adjusted across the four genes using the Holm–Šídák method. Adjusted P values were: *HXK1*, P = 0.0181; *CDC19*, P = 0.0940; *PGI1*, P = 0.6812; and *PGK1*, P = 0.4796. *P<0.05, **P<0.01, ***P<0.001, ****P<0.0001.

### 3.5 *adh2Δ/Δ* shows modestly reduced fluconazole susceptibility

We next examined whether the metabolic changes observed in *adh2Δ/Δ* were associated with altered fluconazole response. Broth microdilution testing showed that the fluconazole MIC increased from 0.5 μg/mL in SC5314 to 1 μg/mL in *adh2Δ/Δ*, indicating a two-fold increase in MIC. Consistently, spot-growth assays showed that *adh2Δ/Δ* retained stronger growth than SC5314 on YPD plates containing 2 μg/mL FLCZ, suggesting enhanced growth under FLCZ stress in the tested condition (**Figure 5A**). At the transcriptional level, *ERG11* remained significantly increased in *adh2Δ/Δ* after correction for multiple testing, whereas *MDR1*, *CDR1*, and *CDR2* were not significantly altered (**Figure 5B**). *ADH1* was also not significantly altered (**Figure 5C**). These results indicate that the modestly reduced fluconazole susceptibility of *adh2Δ/Δ* was accompanied by increased *ERG11* transcript levels but not by significant transcriptional upregulation of the major efflux-associated genes examined.

**Figure 5:**
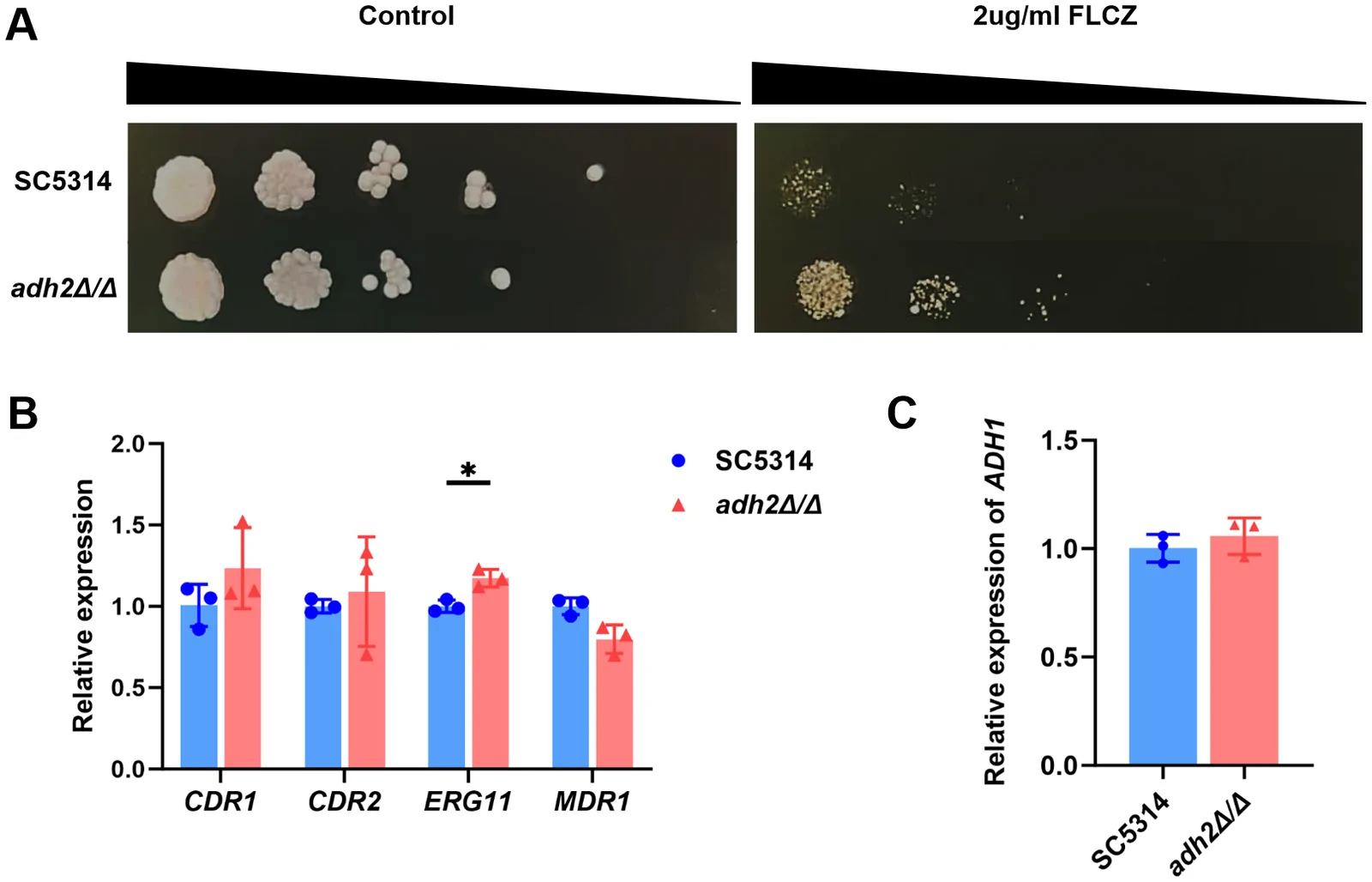
The *adh2Δ/Δ* mutant displays modestly reduced fluconazole susceptibility in *Candida albicans.* **(A)** Spot-growth on YPD control plates and YPD plates containing 2 μg/mL FLCZ; **(B)** relative expression levels of the canonical azole resistance-associated genes *ERG11*, *MDR1*, *CDR1*, and *CDR2*. Expression of each gene was normalized independently to the corresponding SC5314 control. Between-group comparisons were performed using unpaired two-tailed Student’s t-tests, with P values adjusted across the four genes using the Holm–Šídák method (*ERG11*, adjusted P = 0.0413; *MDR1*, adjusted P = 0.0767; *CDR1*, adjusted P = 0.4103; *CDR2*, adjusted P = 0.6691); **(C)** relative expression level of the alcohol dehydrogenase gene *ADH1*. *P<0.05.

### 3.6 *adh2Δ/Δ* is associated with increased renal fungal burden and higher residual fungal burden after FLCZ treatment *in vivo*

Finally, we evaluated *in vivo* infection burden and FLCZ treatment response in the tested immunosuppressed mouse model, with three mice per group used for renal fungal-burden and histopathological analyses and five mice per group used for survival analysis (**Figure 6A**). Gross kidney inspection showed more obvious renal lesions in mice infected with *adh2Δ/Δ* than in those infected with SC5314. Kidney homogenate plating showed greater fungal recovery from the *adh2Δ/Δ* infection group (**Figure 6B**). Kidney-weight-normalized fungal-burden analysis showed that mice infected with *adh2Δ/Δ* had a higher renal fungal burden, expressed as log10 CFU/g kidney, than mice infected with SC5314 (**Figure 6C**). FLCZ treatment markedly reduced renal fungal burden in both infection groups; however, the residual fungal burden remained higher in the *adh2Δ/Δ* + FLCZ group than in the SC5314 + FLCZ group. In the separate survival cohort, mice infected with *adh2Δ/Δ* showed poorer survival than those infected with SC5314, whereas FLCZ treatment prolonged survival in both infection groups (**Figure 6D**). Because renal fungal burden and survival were evaluated in separate subsets of animals, these findings represent parallel *in vivo* phenotypes and do not establish that the higher renal fungal burden caused the poorer survival. Representative H&E- and PAS-stained kidney sections showed visually more apparent fungal elements and renal tissue involvement in mice infected with *adh2Δ/Δ* (**Figure 6E**). These images were used for qualitative illustration only and were not subjected to quantitative histopathological scoring. These results suggest that the *adh2Δ/Δ* mutant is associated with increased renal fungal burden and a weaker treatment-associated reduction in fungal burden after FLCZ administration in the tested immunosuppressed mouse model.

**Figure 6:**
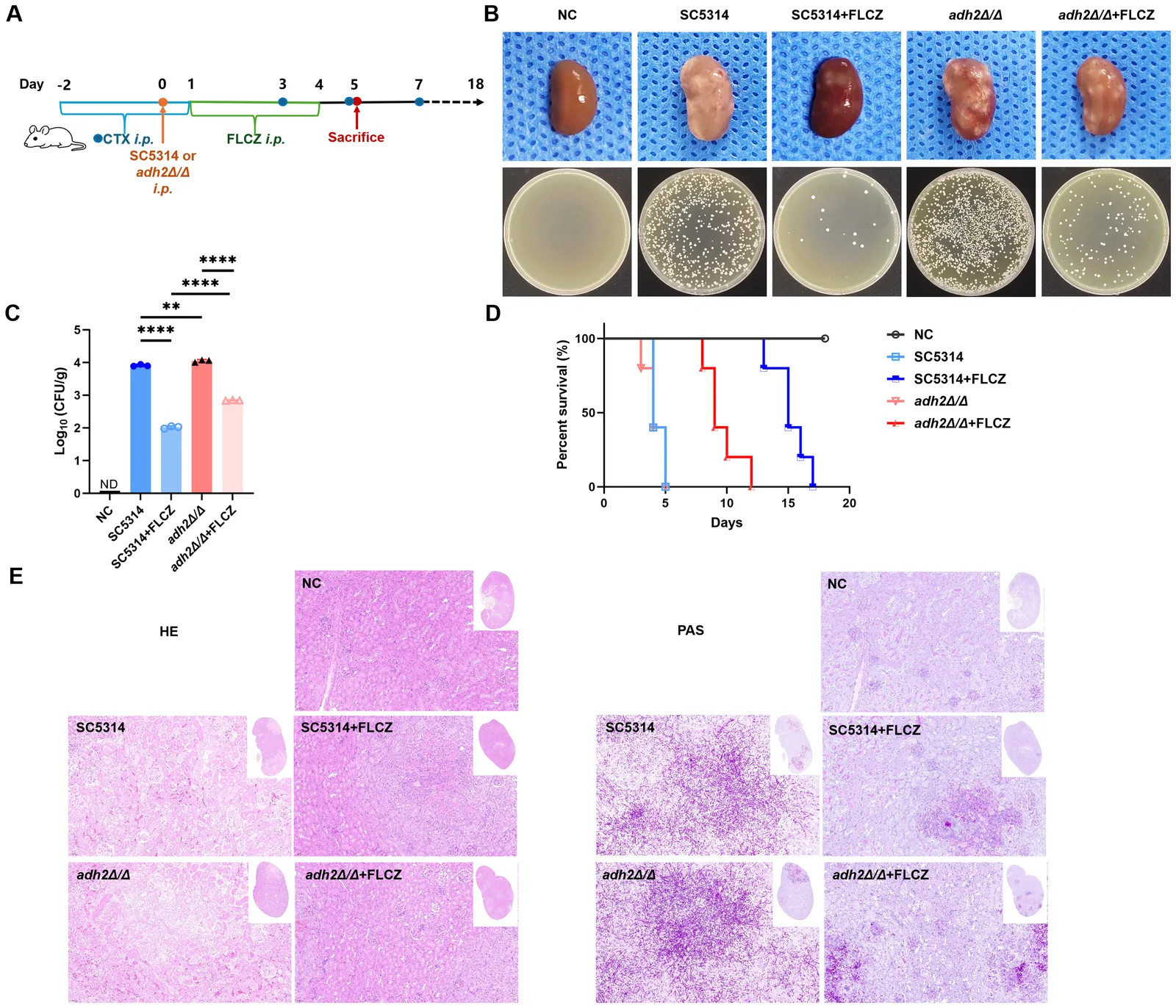
The *adh2Δ/Δ* mutant is associated with increased renal fungal burden and higher residual fungal burden after FLCZ treatment *in vivo*. **(A)** Experimental workflow of immunosuppressed mouse systemic infection and FLCZ treatment; **(B)** Gross kidney morphology and kidney homogenate plating results from different groups; **(C)** Renal fungal burden expressed as log_10_ CFU/g kidney, normalized to the individual kidney weight; **(D)** Survival curves of mice in different groups; **(E)** Representative H&E- and PAS-stained kidney sections from the different treatment groups. Histopathological images were evaluated qualitatively; no quantitative histopathological scoring was performed. NC, normal control; ND, not detected; FLCZ, fluconazole. For renal fungal-burden and histopathological analyses, n = 3 mice per group; for survival analysis, n = 5 mice per group. Renal fungal burdens were analyzed using ordinary one-way ANOVA followed by Tukey’s multiple-comparison test, and survival curves were analyzed using the log-rank (Mantel–Cox) test. **P<0.01, ****P<0.0001.

## 4 Discussion

Compared with *ADH2*, *ADH1* has been relatively more extensively studied among the alcohol dehydrogenase family members in *C. albicans*. Previous work showed that *C. albicans ADH1* is constitutively expressed under multiple growth conditions and that Adh1 contributes to ethanol-utilizing, acetaldehyde-producing activity, whereas the functional contribution of *ADH2* remains less well defined (Bakri et al., 2015). More broadly, fungal alcohol dehydrogenases are involved in carbon utilization, energy metabolism, redox homeostasis, stress adaptation, morphogenesis, and pathogenicity (Gutiérrez-Corona et al., 2023). In *C. albicans*, Adh1 has also been linked to biological activities and pathogenicity-associated traits, and *ADH1* expression has been reported to correlate with azole-resistance-associated genes such as *CDR1*, *CDR2*, and *FLU1* in isolates from patients with vulvovaginal candidiasis (Guo et al., 2013; Wang et al., 2025). However, whether *ADH2* participates in virulence-associated phenotypes and antifungal drug adaptation in *C. albicans* remains unclear. In the present study, by comparing SC5314 with the *adh2Δ/Δ* mutant, we found that *adh2Δ/Δ* did not exhibit a basal growth defect. Instead, this mutant showed modestly altered basal growth kinetics with a shorter doubling time, increased XTT-based biofilm metabolic activity, modestly reduced fluconazole susceptibility, and higher renal fungal burden in the tested immunosuppressed mouse model. At the same time, *adh2Δ/Δ* showed qualitatively reduced adhesion and hyphal development. Together, these findings define a phenotypic profile characterized by divergent changes across virulence-associated traits. These findings suggest a potential association between the *adh2Δ/Δ* phenotype, morphogenesis, metabolic adaptation, and antifungal stress responses in *C. albicans*. Because XTT reduction reflects biofilm-associated metabolic activity rather than total biofilm biomass, the increased XTT signal in *adh2Δ/Δ* should not be interpreted as direct evidence of increased biofilm formation. Moreover, given the altered ATP levels and redox state observed in *adh2Δ/Δ*, the increased XTT signal may also partly reflect altered cellular metabolic reduction capacity rather than increased biofilm biomass. The coexistence of reduced adhesion and cell surface hydrophobicity with increased XTT-based biofilm metabolic activity suggests that surface-associated adhesion properties and biofilm-associated metabolic reduction capacity may be uncoupled in the *adh2Δ/Δ* mutant. Interestingly, *CSH1* expression was modestly increased despite the reduction in measured cell surface hydrophobicity. This discordance indicates that *CSH1* transcript abundance alone does not necessarily predict the overall hydrophobic surface phenotype under the tested conditions. The hydrocarbon-partitioning assay reflects the integrated physicochemical properties of the fungal cell surface, whereas RT-qPCR measures the abundance of a single associated transcript. Thus, increased *CSH1* expression should be interpreted as an associated transcriptional response rather than as a mechanistic explanation for the reduced hydrophobicity. Other cell-surface determinants, post-transcriptional regulation, and condition-dependent surface remodeling may also contribute to this phenotype. Notably, discordance between *CSH1* expression and measured cell surface hydrophobicity has also been reported in a *C. albicans ADH1* deletion mutant, in which increased cell surface hydrophobicity was accompanied by decreased *CSH1* expression. However, because XTT is influenced by cellular metabolic activity, further biomass-based and microscopy-based biofilm assays will be required to determine whether biofilm mass or architecture is altered.

Adhesion and hyphal development are classical virulence attributes of *C. albicans* (Lopes and Lionakis, 2022). The agglutinin-like sequence (ALS) family and hyphal wall protein 1 (Hwp1) are important adhesion- and hypha-associated factors that contribute to interactions with host tissues, abiotic or biomaterial surfaces, and biofilm architecture (Rosiana et al., 2021; Lopes and Lionakis, 2022; Martorano-Fernandes et al., 2023). In the present study, the decreased expression of *ALS1*, *ALS3*, and *HWP1* in *adh2Δ/Δ* was consistent with the qualitative adhesion and filamentation phenotypes observed in the mutant. Because the adhesion assay was based on representative visual comparison rather than direct biomass or CFU quantification, the adhesion phenotype should be interpreted together with the transcriptional changes in *ALS1* and *ALS3*. However, the outcome of infection *in vivo* is not determined by a single hyphal phenotype alone. Instead, it can be shaped by multiple factors, including growth fitness, metabolic state, oxidative stress adaptation, fluconazole response, and host immune clearance (Costa-de-Oliveira and Rodrigues, 2020; Dunker et al., 2021; Lopes and Lionakis, 2022; Pellon et al., 2022). Notably, previous work has shown that a filamentation-defective *C. albicans* strain can retain full virulence through rapid proliferation and improved metabolic adaptation, supporting the concept that reduced filamentation does not necessarily predict attenuated virulence *in vivo* (Dunker et al., 2021). In the tested immunosuppressed mouse model, *adh2Δ/Δ* infection was associated with both higher renal fungal burden and poorer survival. However, these outcomes were evaluated in separate subsets of animals, and no individual-level correlation between renal fungal burden and survival was performed. Therefore, these findings should be interpreted as parallel *in vivo* phenotypes rather than evidence that increased renal fungal burden directly caused poorer survival.

As an annotated alcohol dehydrogenase family member, *ADH2* may be linked to ethanol/acetaldehyde interconversion and NAD^+^/NADH balance, although the direct enzymatic activity of *C. albicans* Adh2 remains incompletely defined (Bakri et al., 2015; Gutiérrez-Corona et al., 2023). In this study, *adh2Δ/Δ* exhibited decreased ATP levels, an increased NAD^+^/NADH ratio, lower basal ROS-sensitive fluorescence, and increased mitochondrial membrane potential. These findings indicate altered metabolic and redox status rather than mechanistically defined mitochondrial dysfunction. In particular, increased mitochondrial membrane potential is not by itself evidence of mitochondrial dysfunction, and reduced ATP levels do not establish that mitochondria are the direct source of the ATP phenotype. Additional mitochondrial functional measurements, such as oxygen-consumption analysis, respiratory capacity, mitochondrial ATP contribution, respiratory-complex activity, or mitochondrial morphology, will be required to determine whether mitochondrial function is directly altered in the *adh2Δ/Δ* mutant. The increased NAD^+^/NADH ratio indicates an altered intracellular redox state in the *adh2Δ/Δ* mutant. Although it is biologically plausible that *ADH2* deletion may affect NAD^+^/NADH balance through alcohol/acetaldehyde-associated metabolism, the present data do not establish this biochemical route. Direct Adh2 enzymatic assays, ethanol/acetaldehyde measurements, and analyses of NAD^+^/NADH metabolism under relevant carbon-source conditions will be required to test this possibility. Together, these findings indicate that the *adh2Δ/Δ* mutant does not simply exhibit enhanced or suppressed energy metabolism, but is associated with altered metabolic and redox status. In particular, the coexistence of increased mitochondrial membrane potential and decreased ATP levels suggests a potential mismatch between mitochondrial electrochemical status and energy production efficiency. Such a phenotype could theoretically arise when maintenance of the proton-motive force is not efficiently translated into ATP synthesis, for example because of altered coupling between electron transport and ATP production or altered proton utilization. However, because respiratory flux, ATP synthase activity, and mitochondrial ATP production were not directly measured, these possibilities remain speculative. Mitochondrial function and redox homeostasis are closely linked to azole susceptibility in *Candida* species, and altered respiratory pathways can affect azole responses, partly through changes in intracellular ROS production (Yan et al., 2009; Song et al., 2020). However, the relationship between azoles, ROS production, and fungal cell fate is complex, and the functional consequences of antifungal drug-induced ROS remain incompletely understood (Gonzalez-Jimenez et al., 2023). Therefore, the reduced ROS-sensitive fluorescence observed in *adh2Δ/Δ* should currently be interpreted as evidence of altered basal redox status rather than direct evidence of reduced oxidative stress during fluconazole exposure, because ROS was not measured under FLCZ treatment in the present study. Further analysis of selected glucose-metabolism-related genes showed that, after correction for multiple testing, *HXK1* remained significantly upregulated, whereas *CDC19*, *PGI1*, and *PGK1* were not significantly altered. The relatively large variability observed for *PGK1* further limits interpretation of this transcript and does not support a consistent change in *PGK1* expression under the tested conditions. Previous studies have shown that sugar phosphorylation and central carbon metabolism influence carbon source utilization, virulence, stress adaptation, and antifungal responses in *C. albicans* (Wijnants et al., 2020, Wijnants et al., 2022). Thus, the significant upregulation of *HXK1*, together with the absence of consistent changes in the other examined genes after multiple-testing correction, supports selective rather than generalized glucose-metabolism-related transcriptional remodeling. Collectively, these results suggest that the *adh2Δ/Δ* phenotype is associated with altered fluconazole response and may involve changes in redox homeostasis, mitochondrial status, and selective glucose-metabolism-related transcriptional changes.

Fluconazole resistance in *C. albicans* commonly involves alterations or overexpression of *ERG11*, which encodes lanosterol 14α-demethylase, the molecular target of azoles, as well as increased drug efflux mediated by ATP-binding cassette (ABC) transporters such as Cdr1 and Cdr2 and the major facilitator superfamily (MFS) transporter Mdr1 (Lee et al., 2021; Maheronnaghsh et al., 2022; Rogers et al., 2022; Lee et al., 2023). By contrast, fluconazole susceptibility and adaptive survival may also involve stress-response pathways and metabolic remodeling, and therefore should not be considered identical to genetically stable resistance (Lee et al., 2021, Lee et al., 2023). CLSI M27 broth microdilution testing showed a two-fold increase in fluconazole MIC in *adh2Δ/Δ* compared with SC5314, supporting a modest reduction in fluconazole susceptibility. However, this modest MIC shift should not be interpreted as breakpoint-defined fluconazole resistance. The transcriptional analyses performed in this study do not establish a molecular mechanism for the reduced fluconazole susceptibility of *adh2Δ/Δ*. *ERG11*, *MDR1*, *CDR1*, and *CDR2* were assessed only at the transcript level, whereas Erg11 protein abundance, sterol composition, target engagement, and transporter activity were not directly measured. After correction for multiple testing, *ERG11* remained significantly upregulated, whereas *MDR1*, *CDR1*, and *CDR2* were not significantly altered. Thus, the reduced fluconazole susceptibility phenotype was not accompanied by significant transcriptional upregulation of the major efflux-associated genes examined. These findings indicate that the altered fluconazole response of *adh2Δ/Δ* was associated with increased *ERG11* transcription, but they do not establish that *ERG11* mediates this phenotype. Direct Erg11 protein measurement, ergosterol content analysis, azole target-engagement assays, and *ERG11* functional manipulation would be required to test whether *ERG11* contributes causally to the reduced fluconazole susceptibility observed in *adh2Δ/Δ*. Notably, after correction for multiple testing, *HXK1* remained significantly upregulated in *adh2Δ/Δ*, whereas *CDC19*, *PGI1*, and *PGK1* were not significantly altered. Thus, the altered fluconazole response coexisted with selective glucose-metabolism-related transcriptional changes rather than generalized glycolytic activation. However, because glucose consumption, ethanol production, pyruvate- or lactate-related metabolites, oxygen consumption, and intracellular metabolomics were not assessed, these transcript-level findings should not be interpreted as direct evidence of altered glycolytic flux.

Taken together, our findings support a three-domain model of the *ADH2* deletion-associated state in *C. albicans*. First, *adh2Δ/Δ* displayed altered cellular phenotypes, including modestly altered growth kinetics, reduced adhesion and filamentous development, increased XTT-based biofilm metabolic activity, and reduced cell surface hydrophobicity. Second, the mutant exhibited an altered metabolic/redox state characterized by reduced ATP levels, decreased basal ROS-sensitive fluorescence, an increased NAD^+^/NADH ratio, elevated mitochondrial membrane potential, and selective changes in glucose-metabolism-related transcript levels. Third, *adh2Δ/Δ* showed modestly reduced fluconazole susceptibility accompanied by increased *ERG11* transcript levels, together with higher renal fungal burden and poorer survival in the tested immunosuppressed mouse model. These domains were evaluated independently, and the present data do not establish a linear causal hierarchy among them. Rather, they define a coordinated *ADH2* deletion-associated phenotype and provide a hypothesis-generating framework for future mechanistic studies examining how metabolic/redox changes may relate to fungal adaptation and fluconazole response.

Several limitations of this study should be acknowledged. Most importantly, the study lacks an *ADH2* reintegrant strain and functional validation using independently constructed homozygous deletion clones. Therefore, although the *adh2Δ/Δ* mutant displayed changes in growth, adhesion, filamentation, biofilm metabolic activity, redox status, glucose-metabolism-related gene expression, fluconazole response, and renal fungal burden, these findings cannot by themselves establish that *ADH2* loss is the sole cause of all observed phenotypes. Secondary genetic changes introduced during strain construction, including potential off-target changes, aneuploidy, or loss of heterozygosity, as well as other background-specific effects, may have contributed to some of the observed phenotypic differences. In addition, adhesion and filamentation were assessed mainly by representative visual observations supported by *ALS1*, *ALS3*, and *HWP1* expression analysis, but were not further validated by quantitative adhesion assays or morphological measurements. For this reason, the current study should be interpreted as an exploratory phenotypic and metabolic characterization of an *ADH2* deletion-associated state. Future work using genetic complementation, independently generated deletion mutants, and direct Adh2 enzymatic assays, ethanol/acetaldehyde measurements, and carbon-source-specific NAD^+^/NADH analyses will be required to confirm the causal contribution of *ADH2* to these processes.

Despite these limitations, the present study identifies a coordinated *ADH2* deletion-associated phenotype spanning cellular, metabolic/redox, antifungal-response, and *in vivo* infection-related domains. Rather than establishing a single mechanistic pathway, these findings provide a structured hypothesis-generating framework for determining which of these associations are causally linked in future studies.

## Data Availability

The original contributions presented in the study are included in the article/**Supplementary Material**. Further inquiries can be directed to the corresponding authors.
