# Supplementary material for: Phenotypic and metabolic characterization of an ADH2 deletion mutant associated with reduced fluconazole susceptibility in Candida albicans
Source: Front Cell Infect Microbiol. 2026 Oct 9;16:1911561. doi: 10.3389/fcimb.2026.1911561 (PMC13690679; doi:10.3389/fcimb.2026.1911561)
Supplement: Supplementary file 1 [file Table1.docx]

**Table S1. *Candida albicans* strains constructed to study the role of *ADH2* (orf19.5113).**

| **Label used in manuscript** | **Actual strain/project ID** | **Description** | **Parent/background** | **Phenotype** | **Genotype** |
| --- | --- | --- | --- | --- | --- |
| SC5314 (SC5314-background parental control) | SN152 | Parental/control strain used for ADH2 deletion | SC5314-derived | Arg− Leu− His− | arg4Δ/arg4Δ leu2Δ/leu2Δ his1Δ/his1Δ URA3/ura3Δ::imm434 IRO1/iro1Δ::imm434 |
| adh2Δ/ADH2 | S1 | Heterozygous ADH2 deletion mutant | SN152 | Arg− His− | adh2Δ::C.m.LEU2/ADH2 arg4Δ/arg4Δ leu2Δ/leu2Δ his1Δ/his1Δ URA3/ura3Δ::imm434 IRO1/iro1Δ::imm434 |
| adh2Δ/Δ | D1 | Homozygous *ADH2* deletion mutant used for phenotypic and functional assays | S1 | Adh2− Arg− | adh2Δ::C.m.LEU2/adh2Δ::C.d.HIS1 arg4Δ/arg4Δ leu2Δ/leu2Δ his1Δ/his1Δ URA3/ura3Δ::imm434 IRO1/iro1Δ::imm434 |

Note: The parental strain used for genetic manipulation was SN152, an auxotrophic strain derived from the SC5314 genetic background. For consistency with the main text and figures, the parental control is referred to as the SC5314-background parental strain.

**Text S1. Construction of *ADH2* deletion strains.**

*ADH2* deletion in *Candida albicans* (*C. albicans*) was performed using fusion PCR and homologous recombination based on a previously described *C. albicans* genetic manipulation strategy. The parental strain used for genetic manipulation was SN152, an auxotrophic strain derived from the SC5314 genetic background. Genomic DNA was extracted from the parental strain using the Yeast Genomic DNA Extraction Kit according to the manufacturer’s instructions.

The upstream and downstream homologous arms of the *ADH2* open reading frame were amplified using primer pairs P1/P3 and P4/P6, respectively. Nutritional selection marker fragments were amplified from pSN40 and pSN52 using primers P2/P5. The purified PCR fragments were assembled by fusion PCR to generate homologous recombination cassettes containing the *ADH2* flanking regions and nutritional markers. The first *ADH2* allele was replaced with the *C.m.LEU2* cassette to generate the heterozygous deletion strain S1, designated *adh2Δ/ADH2*. The second *ADH2* allele was replaced with the *C.d.HIS1* cassette to generate the homozygous deletion strain D1, designated *adh2Δ/Δ*. Primer sequences and PCR conditions are provided in Tables S2 and S3.

**Table S2. Primers used for construction of *ADH2* deletion cassettes.**

| Primer | | Sequence (5′ to 3′) | Function | Position |
| --- | --- | --- | --- | --- |
| P1 | AACATCTTTCATCATCATTCATCC | | Used for amplifying the 5’ homologous recombination arm | Forward primer, located at position 515 of *ADH2* 5’ UTR |
| P3 | cacggcgcgcctagcagcggTGTAATTGAGTTAGATTTAATAAAAAGTG | |  | The uppercase sequence is the reverse primer, located at position 3 of *ADH2* 5’UTR. The lowercase sequence is a linker sequence, unrelated to the ADH2 sequence. |
| P4 | gtcagcggccgcatccctgcGCGCATTCATGCCAAGTAC | | Used for amplifying the 3’ homologous recombination arm | The uppercase sequence is the forward primer, located at position 1 of *ADH2* 3’ UTR. The lowercase sequence is a linker sequence, unrelated to the *ADH2* sequence. |
| P6 | TGTGTAGTTGATTTATAATAATAGCG | |  | Reverse primer, located at position 564 of *ADH2* 3’ UTR |
| P2 | ccgctgctaggcgcgccgtgACCAGTGTGATGGATATCTGC | | Used for amplifying the marker | Unrelated to the *ADH2* sequence; the lowercase sequence is a linker sequence |
| P5 | gcagggatgcggccgctgacAGCTCGGATCCACTAGTAACG | |  |  |

Lowercase letters indicate linker sequences used for fusion PCR; uppercase letters indicate gene- or marker-specific sequences.

**Table S3. PCR cycling conditions.**

| Step | Temperature (°C) | Time |
| --- | --- | --- |
| Initial denaturation | 94℃ | 2min |
| Cycle (35×) | 94℃ | 30s |
|  | 52℃ | 50s |
|  | 68℃ | 8min |
| Final extension | 68℃ | 15min |

Fig. S1 Construction of *ADH2* (orf19.5113) homologous recombination cassette.

The *C.m.LEU2* deletion cassette was first introduced into the SC5314-background parental strain SN152 using the lithium acetate transformation method to generate the heterozygous deletion strain S1. The *C.d.HIS1* deletion cassette was subsequently introduced into S1 to generate the homozygous deletion strain D1. Colonies were verified by diagnostic PCR.

Fig. S2 Strategy for *ADH2* gene deletion via homologous recombination.

**Text S2. Verification of *ADH2* deletion strains**

Diagnostic PCR was used to verify replacement of the *ADH2* locus by the homologous recombination cassettes. The internal *ADH2* primer pair Oligo-1/Oligo-2 was used to detect the presence of the *ADH2* open reading frame. The distal flanking primers Oligo-3/Oligo-4 and marker-specific primers Oligo-M1/Oligo-M2 and Oligo-M3/Oligo-M4 were used to verify correct integration of the *C.m.LEU2* and *C.d.HIS1* cassettes. Primer sequences are listed in Table S4, and the verification strategy is shown in Fig. S3.

Fig. S3 Diagnostic PCR strategy for verification of *ADH2* deletion. Positions of the internal *ADH2* ORF primers, distal flanking primers, and marker-specific primers used for verification are shown.

**Table S4. Primers used for verification of *ADH2* deletion strains.**

| Primer | Primer sequence | Function | Position |
| --- | --- | --- | --- |
| Oligo-1 | ACGGTTCTTGTTTGAACTGTG | Amplifies a segment in the *ADH2* ORF | Forward primer, located at position 284 of the *ADH2* ORF |
| Oligo-2 | TGATAACACCATGTGGACCAC |  | Reverse primer, located at position 733 of the *ADH2* ORF |
| Oligo-3 | TTTTCAAGCTAAGGAGTTTAAACC | Distal primer for 5’UTR | Forward primer, located at position 539 of *ADH2* 5’UTR |
| Oligo-4 | AGACAGAGCTTCTAAGCAG | Distal primer for 3’UTR | Reverse primer, located at position 590 of *ADH2* 3’UTR |
| Oligo-M1 | AGAATTCCCAACTTTGTCTG | *LEU2* Check Left | Tag primer, unrelated to the *ADH2* sequence |
| Oligo-M2 | AAACTTTGAACCCGGCTGCG | *LEU2* Check Right |  |
| Oligo-M3 | ATTAGATACGTTGGTGGTTC | *HIS1* Check Left |  |
| Oligo-M4 | AACACAACTGCACAATCTGG | *HIS1* Check Right |  |

**Text S3. Validation results of *ADH2* deletion strains.**

In the heterozygous deletion strain S1, diagnostic PCR confirmed correct replacement of one *ADH2* allele with the *C.m.LEU2* cassette. In the homozygous deletion strain D1, no amplification product was detected using the internal *ADH2* ORF primer pair Oligo-1/Oligo-2, whereas the expected marker-junction PCR products were detected. These results confirmed replacement of both *ADH2* alleles in D1, designated *adh2Δ/Δ*. Although additional PCR-positive colonies were obtained during strain construction, only the verified D1 clone was used for phenotypic, metabolic, antifungal-response, and animal experiments in this study.

Fig. S4 PCR verification of the heterozygous *ADH2* deletion strain *adh2Δ/ADH2*.

Fig. S5 PCR verification of the homozygous *ADH2* deletion strain *adh2Δ/Δ*.

**Sequence S1. Genomic sequence of *ADH2* (orf19.5113) used for primer design.** The sequence includes the 5′ UTR, ORF, and 3′ UTR (The yellow-labeled section is the *ADH2* ORF).

TCCACACTTCAAAACAAGCCAAACACTTTTTTGGGCGAGTACACCTCGTCAAATAAGACACAAAATCTAACTGAAAACCTAATCGCATTTATCAAATGGTATTGGATATAGGATAATTCATAATTTAGCTTTTTGGATGATTAGATCATACGGTTGATGTCAACTTTAAGCTTTTATGTTAATCTGGTCGGATGATTTTTTTTTTTTTGCATTTGGGTAGTTTTTAATTATCGGAAAATAATCAGCCGTGGTAAAAATATACCATACAACAGGTAAATGTTGTCAATGGCTAGAGCCAGATAACCAAGAAATAATTAGGAAAATTCATTTGCAATGTTGAACATAATTTCAGTTAAACTAACACAAAAACTACACGATTAATTCATTTACTGCAGAATAACAAACACGGACAACACATATGCACACGAAATCCACTATTCAATTGGGCACCATACACAAATTTTTCAAGCTAAGGAGTTTAAACCAACATCTTTCATCATCATTCATCCAGTGTGGAGAAGCTAATGAAAGAAATGTATGTTGTGCATGCAAAGTAAAAGGAACTACTCCCACACTCCAACTATTAAAAGCACGTTGAATTAAAAAGAGAAATACGCAGTGGAGTATAATACACCAAGTAAATAGTTCTGTTTTGTGTCACAATCTGGAGCACCCCCACCGACTCCGCTGCTGCTGCTCCTCATCCATCTTATTCGCTGGGCGTTCAATTTTTTCATTCTCTCTGCTATCTCCTTCCCCCCTAATCTCCCATGTACATGGATCACTCTTGCAAGCTAATCTCCTATAATTATTCAGTTTTATAATAAAATATTTTCTTTCCAAAAAGGAAATGAAAAAACTATAAATACCCCTAGTTATTCCTCTATTTTCATCACTAAATTTTCATTCAATTGTTTTAAACTATCAATCAATTGTTCATCTATAATCACGAATCAATTGATACTTACCCACTTTTTATTAAATCTAACTCAATTACACCATGTCTGTCCCAACTACTCAAAAAGCTGTTATTTTCGAAACCAACGGTGGTAAGTTGGAATACAAAGATATCCCAGTCCCAAAACCAAAAGCAAATGAATTGTTAATCAACGTCAAATACTCTGGTGTCTGTCACACTGATTTACACGCTTGGAAAGGTGACTGGCCATTGGCTACTAAATTGCCATTGGTTGGTGGTCACGAAGGTGCTGGTGTTGTTGTCGCCTTGGGTGAAAACGTTAAAGGCTGGAAAGTTGGTGATTACGCTGGTGTTAAATGGTTGAACGGTTCTTGTTTGAACTGTGAATACTGTCAATCAGGTGCTGAACCAAACTGTGCTGAAGCTGATTTATCCGGTTACACCCACGATGGTTCTTTCCAACAATATGCTACTGCTGACGCTGTTCAAGCTGCCAGAATCCCAGCTGGTACCGACTTAGCCAATGTTGCACCAATCTTATGTGCTGGTGTCACCGTCTACAAAGCTTTAAAGACTGCTGAATTAGAAGCCGGTCAATGGGTTGCTATTTCCGGTGCCGCTGGTGGTTTAGGTTCTTTAGCTGTTCAATACGCCAAGGCCATGGGTTACAGAGTTCTTGCCATCGATGGTGGTGAAGACAAGGGTGAATTCGTCAAATCCTTGGGTGCTGAAACCTTTATTGATTTTACCAAAGAAAAAGACGTTGTCGAAGCTGTCAAGAAGGCCACCAATGGTGGTCCACATGGTGTTATCAATGTCTCTGTCTCAGAAAGAGCCATTGGTCAATCCACTGAATATGTCAGAACTTTAGGTAAAGTTGTTTTGGTTGGTTTGCCAGCTGGTGCTAAAATTAGTACCCCAGTCTTTGATGCTGTTATCAAGACCATTCAAATTAAAGGTTCTTATGTCGGTAACAGAAAAGATACTGCTGAAGCCGTTGATTTCTTCACAAGAGGTTTGATCAAATGTCCAATCAAGATTGTTGGCTTATCCGAATTACCAGAAGTTTACAAATTGATGGAAGAAGGTAAAATCTTGGGTAGATATGTCTTGGACAACGACAAATAAGCGCATTCATGCCAAGTACGATAATAATGAGTGATGAAGACGACGACTAAAATAAATAAATAATAACAACAGATGTGTTTGCTTTTTTATTGAGTAAGTTTAAGCTAAACGCATTTGAGAGTTTGATACTCTGATGTTATTTGTTATATTTTTTTTTTTATAGTTAGTGTCTAGAATTTTAGTCTTTCAATACAGAATTGATAAATTTTTGAGTTGTTTTCTCTGAGGTGATATTATTGGGTTTATGAAGAAGGTGGTTATTCCAAGCTCTTGATATAGAATTTGTTCTGACTTTTTGCAACAAAAAAAAGGAGTTCATATTTGGCTCATGATCCTAATGGAGCAAAAATGGACCAAGCCAGGTATGGTCTATATGTCAACACTCCTTCGTAATCGATGACTTAAAGAACAGCGTTTGCGATAGAACAGCTTAGTATCCTAAATCAAGCTGTTCTGTAGTTAGAGTCTTGAGCAATACCACTATAGGCTTTGACTACAAAGTTAGATAGAAAACTATCACTAAATAAAAAAAATGTATCGCTATTATTATAAATCAACTACACACTACCTCTGCTTAGAAAGCTCTGTCTAAAGGAGACAACAATTGTTTACCTTCGTAATATTTAATAGTCTTTTGAGCTCTTTCGGCAATTCCTTCTGGAGTAAACTCAAAGTACTTGTACAAATCAGCAGCCTTACCTGAAGCACCAAATCTGTTCAAACCAAATTGTTCATGAGAGTATTTAGACCATCCGAAGGTGGACATGACTTCAACAGACAAGATTGGAACACCATCTGGGAGAACTGACAATCTATATTCATCAGATTGTTGGTCGAAGGTGAAAAAGTCTGGCAAGGAAACAACATTAGCCTTAACTCCTTGTTTAGCTAATTCTTCACTAGCAGCAACAGAAATTGAAACTTCAGAACCAGAAGAAACAATAATAACGTCTGGGTTGTCTTTCTTGACAAGAGTGTAACCACCCTTCAAGGCGTTTTC

**Table S5. Primers for RT-qPCR.**

| Primers name | Sequence (5’ to 3’) |
| --- | --- |
| 18SrRNA-F | GTGCCAGCAGCCGCGGTA |
| 18SrRNA-R | TGGACCGGCCAGCCAAGC |
| ALS1-F | TTGGGTTGGTCCTTAGATGG |
| ALS1-R | ATGATTTCAAAGCGTCGTTC |
| ALS3-F | ACTAAAGGAAACAACGGAAATG |
| ALS3-R | AGAAATGATAGGCGATGAAGC |
| CSH1-F | CTGTCGGTACTATGAGATTG |
| CSH1-R | GATGAATAAACCCAACAACT |
| HWP1-F | CACAGGTAGACGGTCAAGGT |
| HWP1-R | AAGGTTCTTCCTGCTGTTGT |
| ERG11-F | TTGGTGGTGGTAGACATAGATG |
| ERG11-R | AACTATAATCAGGGTCAGGCAC |
| MDR1-F | AGTTGCTTGGGGTAGTTCCG |
| MDR1-R | CTTGCTCTCAACTTTGGTCCG |
| CDR1-F | TGTTGGGTTGGTCTCGATG |
| CDR1-R | TCATAACCTGGACCACTTGG |
| CDR2-F | ATGCCAATGCTGAACCGAC |
| CDR2-R | AAAGTTGTAGCCAAATTAGCAGC |
| CDC19-F | GCTATCTTGGATGGTGCTGACTG |
| CDC19-R | GCATCATAGAGACGGCTTCAACTG |
| PGK1-F | GTGGTGGTGATACTGCTACTGTTG |
| PGK1-R | CTACACCTGGCAAGTCTTTACCTTC |
| PGI1-F | GATGGGAAAGATACCGCTCAAGAAG |
| PGI1-R | GCCAGTGTAACCAGTCCAAGAAC |
| HXK1-F | TGACGAGGTTGGTGGTGGTAG |
| HXK1-R | CTCTTGGTACATCTGTTGGTGGTC |
| ADH1-F | CACTCACGATGGTTCATTCG |
| ADH1-R | AAGATGGTGCGACATTGG |
